# Evaluating the Role of Attention in the Context of Unconscious Thought Theory: Differential Impact of Attentional Scope and Load on Preference and Memory

**DOI:** 10.3389/fpsyg.2013.00037

**Published:** 2013-02-04

**Authors:** Narayanan Srinivasan, Sumitava Mukherjee, Maruti V. Mishra, Smriti Kesarwani

**Affiliations:** ^1^Centre of Behavioural and Cognitive Sciences, University of AllahabadAllahabad, India

**Keywords:** decision making, preference, memory, global attention, local attention, unconscious thought, distraction

## Abstract

Attention is a key process used to conceptualize and define modes of thought, but we lack information about the role of specific attentional processes on preferential choice and memory in multi-attribute decision making. In this study, we examine the role of attention based on two dimensions, attentional scope and load on choice preference strength and memory using a paradigm that arguably elicits unconscious thought. Scope of attention was manipulated by using global or local processing during distraction (Experiment 1) and before the information-encoding stage (Experiment 2). Load was manipulated by using the n-back task in Experiment 1. Results from Experiment 1 show that global processing or distributed attention during distraction results in stronger preference irrespective of load but better memory only at low cognitive load. Task difficulty or load did not have any effect on preference or memory. In Experiment 2, distributed attention before attribute encoding facilitated only memory but did not influence preference. Results show that attentional processes at different stages of processing like distraction and information-encoding influence decision making processes. Scope of attention not only influences preference and memory but the manner in which attentional scope influences them depends on both load and stage of information processing. The results indicate the important role of attention in processes critical for decision making and calls for a re-evaluation of the unconscious thought theory (UTT) and the need for reconceptualizing the role of attention.

## Introduction

Life is mostly about choices. From choosing to buy a car or a chocolate to a house or a pen, choices are diverse. A large number of studies on decision making assume that cognition involves two hypothesized modes of thought (Sloman, 2002; Kahneman, 2011) – a fast, less controlled, and intuitive System 1 and a slow, controlled, and deliberate System 2 (Stanovich and West, 2002; Kahneman, 2011). Deliberation requires more cognitive and attentional resources but is classically thought to be more dependable for complex decisions (Christoff et al., 2011). Some have argued that the two modes of intuition and deliberation underlie a continuum in decision making (Hammond et al., 1987). These modes might be required and used according to the problem at hand (Ariely and Norton, 2011). For instance, deciding in situations when self-protection or sexual jealously is prominent requires too little thinking, while choosing a job or car requires deliberation. In many demanding naturalistic contexts however, intuitive decisions yield significant advantages wherein people use prior experience to rapidly categorize situations (e.g., Klein, 2008). In many real life situations like managerial decision making, neither thinking too much nor thinking too little is advantageous. In fact most managers err in choosing one over the other (Ariely and Norton, 2011).

Contrary to classical wisdom, Unconscious Thought Theory (UTT) proposes that compared to conscious careful deliberation, normatively better decisions for complex multi-attribute problems are made following “unconscious thought” (UT; Dijksterhuis, 2004; Dijksterhuis and Nordgren, 2006; Dijksterhuis et al., 2006, 2009). Even the strength of preference measured by subtracting the average rating of the three lower rated options from the one rated highest for the chosen alternative is higher following UT (Dijksterhuis and van Olden, 2006). UTT theorists suggest we should think less and depend on the powerful unconscious for making complex decisions (Dijksterhuis, 2004). Such exciting messages of clearly opting for “thinking too little” and asking the “unconscious to deal with it” must be treated with caution and require much more clarification. For instance, it is not clear what is actually meant by “unconscious” in this context.

According to UTT, unconscious thought (UT) is defined as object-relevant or task-relevant cognitive or affective thought processes that occur while attention is directed elsewhere (Dijksterhuis and Nordgren, 2006). In a typical UTT paradigm, at first, information is presented in the encoding stage. This is followed by a period of conscious, unconscious, or immediate thought. Conscious thought involves carefully attending to the problem and deliberating over it, while unconscious thought is elicited when distracted by an unrelated task. Immediate thought occurs when one has to decide immediately after the information is presented. During distraction, attention is supposed to be *directed away* from the problem, resulting in deliberation-without-attention.

Although some studies have found supportive evidence for the superiority of unconscious thought (e.g., Ham et al., 2009; Usher et al., 2011), quite a few attempts to replicate the unconscious thought effect have failed (Lerouge, 2009; Newell et al., 2009; Rey et al., 2009; Thorsteinson and Withrow, 2009; Waroquier et al., 2010; Newell and Rakow, 2011). Some have also argued for alternate explanations (Calvillo and Penaloza, 2009; Lassiter et al., 2009; Srinivasan and Mukherjee, 2010). Conflicting evidence has resulted in controversies on the advantages of intuition versus deliberation (Kahneman and Klein, 2009) and unconscious versus conscious modes of thought (Newell and Rakow, 2011).

However, note that in all these debates, the primary concern is whether unconscious mode of thought is superior to conscious deliberation. None of these studies discuss the nature of processes like attention *during* the unconscious thought. Hence, in order to understand the role of attention in the decision making process, it is necessary to go beyond whether attention is present or absent and which mode of thought is normatively superior. Although UT theorists claim that attention is directed elsewhere during a distracter task, it remains to be determined if different types of attentional processes employed in various modes of thought or during distraction matter. Additionally, our later responses and memory can also be influenced by the stages of processing (such as information presentation versus distraction) at which different attentional mechanisms could be employed. Could there be differences in attentional mechanisms based on the type of distraction task? Can the results be influenced by the type and timing of attentional strategies employed? Can attentional processes modulate other mechanisms such as preference or memory?

Attention is a crucial cognitive process that affects preferences and choices (Mukherjee and Srinivasan, 2013). Attention can be qualitatively conceptualized either as a resource weighted as more or less (Kahneman, 1973), or as differences in scope rated as narrow or wide (Srinivasan et al., 2009; Förster and Dannenberg, 2010). The choices we make and preferences we form can be biased by manipulating relative visual attention to the alternatives (Armel et al., 2008). However, the role of attentional scope on different stages of decision making (such as pre versus post-information acquisition) remain largely unknown. A survey of the literature reveals that the specific role of attentional processes or manipulations *during* unconscious thought has not been examined to date, and is therefore the focus of our study.

An important aspect of attention is its scope that determines the number of items selected for further processing. Focusing or narrowing attention via local processing versus distributing or widening attention via global processing affects many cognitive and emotional processes differently (Förster and Dannenberg, 2010; Srivastava and Srinivasan, 2010; Srinivasan and Gupta, 2011; Förster and Denzler, 2012b). Global processing has been shown to be associated with a promotion focus with eager approach strategies (Förster and Higgins, 2005), higher liking for atypical objects (Förster and Denzler, 2012a), positive moods (Gasper and Clore, 2002), and better processing of happy faces (Srinivasan and Hanif, 2010; Srinivasan and Gupta, 2011). Measures associated with scope of attention have been linked to filtering abilities of items and working memory capacity (Vogel and Machizawa, 2004; Cowan, 2005). Changes in scope of attention affect memory (Macrae and Lewis, 2002; Cowan, 2005; Srinivasan and Gupta, 2011) and other cognitive processes including intelligence (Cowan et al., 2006). Scope of attention can be manipulated with global or local processing using hierarchical composite stimuli, which consist of a large or global letter made up of identical small or local letters (Navon, 1977). In the global task, participants have to identify the large letter and in the local task they have to identify the small letter.

Attention is also conceived in terms of the amount of resources needed to perform a given task. Tasks vary in terms of difficulty and hence in terms of attentional resources needed to perform the task. Task difficulty has been shown to influence processes associated with judgment and decision making (Sprenger et al., 2011). One way of manipulating task difficulty would be to use tasks that differentially load working memory using the *n*-back tasks (Smith and Jonides, 1998) with the zero-back task loading the cognitive system (working memory) less compared to the two-back task, which require maintaining two items in working memory and updating them on a trial-by-trial basis. This load manipulation can be orthogonally combined with the scope of attention manipulation resulting in four different task conditions.

In the current study, we have investigated the potential role of global versus local attention as well as low versus high load during distraction (hypothesized to generate unconscious thought) on choice preferences and memory. We predicted that the scope of attention used during the distraction period would have a direct bearing on the nature of decisions and memory. We hypothesized that a global task during distraction results in higher strength of preference given its link with promotion focus (Förster and Higgins, 2005) and liking atypical objects (Förster and Denzler, 2012a). If task difficulty of the distraction task is a critical factor and exhausts mental resources, then one would expect better preference with the low load compared to the high load task according to the classical view which regards attentional resources critical to make a complex decision with many informational attributes but not according to UTT (Dijksterhuis and Nordgren, 2006).

Both the distraction task and the encoding stage can potentially affect memory of the choice alternatives. Memory-related processes have been proposed to explain some of the effects associated with UT and play an important role in decision making (Shanks, 2006; Lassiter et al., 2009). Memory may be affected by the type and timing of attentional strategies employed during distraction (UT). In general, consolidation in memory is faster for tasks that involve distributing attention over a large number of items (Baijal and Srinivasan, 2011). Given that items that are attended to, get privileged access (McElree, 2006), selected items become more active in working memory and receive additional processing. Enhanced processing of the selected items should enable people to remember them better. With increase in scope of attention, one can expect more items or attributes to be selected and benefited from additional processing. We therefore predicted that participants performing a global task would have better memory of the information presented compared to those who perform a local task. In terms of load, if attentional resources are not necessary for unconscious thought (distraction) as it does not have limited capacity like conscious thought or most of the encoding is performed online, then load would not affect memory of the attributes. However, if attentional resources (more or less) are critical for processing during distraction, then we would expect load to influence memory such that low load results in better memory compared to the high load condition.

Proponents of UTT further suggest that distraction improves decision quality because UT occurs after information is presented and is off-line (Strick et al., 2010). However, other researchers have suggested that decisions are taken during information presentation in the encoding phase, implying judgments are online (Lassiter et al., 2009) and distraction does not improve decisions (Newell et al., 2009; Waroquier et al., 2010). If decisions are made based only on online information processing, then manipulating attention during the distraction period must not affect strength of preference for the choice. However, even if decisions are taken primarily online, a time interval post-information presentation (regarded as distraction period in UTT) can affect memory of the choice alternatives. The scope of attention can also affect encoding of information itself. Thus, by manipulating the scope of attention both during the distracter task and before the presentation of attribute information, it is possible to examine how decision making and memory processes function both during and after encoding. In experiment 1, we measure the effect of manipulating scope and load during distraction (unconscious thought) after presentation of attribute information and in experiment 2, we investigate the effects of attentional scope before the presentation and encoding of attribute information.

## Experiment 1

Participants were engaged in a global or local task that was either easy (low load) or difficult (high load) during the distraction period when UT was supposed to occur according to UTT. Instead of conflating both attention and consciousness, it is possible to manipulate attention without necessarily manipulating consciousness (Srinivasan and Mukherjee, 2010). This manipulation would also allow us to compare the effects of attentional scope (global versus local processing) as well as task difficulty (low versus high load) during distraction on preferential choice and encoding of information. We expected better preference and memory with global compared to local task during distraction. In addition, if we assume distractor task difficulty would reduce preference and memory then values would be lower with the two-back compared to the zero-back task due to increased interference to memory in a high load task (according to the classical view). Note that according to UTT, one would hypothesize that a more difficult task would result in increase of preference strength as distracting more away from the problem results in better quality of decisions according to UTT theorists. More importantly, differences due to scope or load would imply that attentional strategies employed *during* the distraction period of UT modulate the primary judgment or choice task.

### Method

#### Participants

Eighty nine naïve adults with normal or corrected-to-normal vision from Allahabad, India participated in the experiment. Each person was paid a nominal amount for participating in the study.

#### Stimuli and apparatus

The hierarchical stimuli (Navon, 1977) used in the global task consisted of S, H, 6, and 9 at the global level with eight at the local level. Similarly, the stimuli used in the local task contained S, H, 6, and 9 at the local level with eight at the global level. Global letters subtended 3.7° × 5.5° while local letters subtended 0.35° × 0.65°. Stimuli were presented on a monitor and response was obtained using the key board.

#### Procedure

Participants were provided with four cell phones, namely *X*1, *X*2, *X*3, and *X*4. The decision involved choosing the best cell phone out of the four and rating all of them. Although we were not interested in the normative quality of choice, we asked participants to judge the best cell phone: (i) so that we could measure preference strength and (ii) because unconscious thought is goal-directed (Bos et al., 2008). Also, note that we do not discuss the normative best decision but rather calculate the dependent measures based on the subjective ratings of the participants. Most experimenters in the UTT studies (e.g., Nordgen et al., 2011) ignore subjective idiosyncratic weights (but see Newell et al., 2009; Usher et al., 2011) that have recently been argued to be a major concern for replication of findings based on UTT (Newell and Shanks, in press). Following the paradigm used in UTT experiments, participants were told that they would be provided with 12 attributes of each cell phone. Based on the information, participants would first form an impression about each cell phone, and later rate each of the phones. Presence or absence of each attribute in the four cell phones was tabulated in a row and each attribute row was separately presented to the participants for 15 s each (see Table 1 for the attribute information for the cell phones). The total presentation time for the 12 attributes was 3 min.

**Table 1:** **Dataset used in the study indicating the presence or absence of attributes for all the four cell phones**.

Attribute	*X*1	*X*2	*X*3	*X*4
MP3 player	1	−1	1	−1
Camera zoom	−1	1	−1	−1
FM radio	1	1	1	−1
Track ID	−1	1	−1	−1
Video recording	1	−1	1	1
Email	−1	1	−1	1
Video playback	−1	−1	1	1
GPS	−1	−1	−1	1
MMS	−1	1	1	1
3G	−1	1	1	1
Flight mode	−1	−1	−1	1
TV output	−1	−1	−1	1

**Figure 1:**
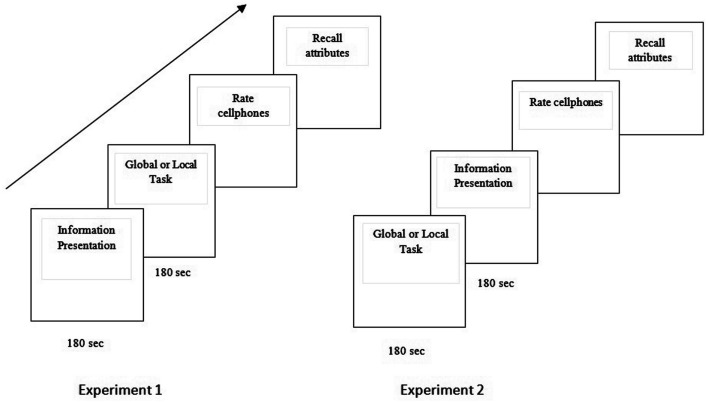
**Block diagram schematic of the order of different stages in Experiment 1 and 2**.

After the information presentation stage, participants were asked to perform either a global or a local task for 3 min and then judge the cell phones (see Figure 1). There were four different between-subjects task conditions: global zero-back, local zero-back, global two-back, and local two-bask conditions. The hierarchical letters were shown consecutively for 500 ms at the center of the screen followed by a blank screen for 500 ms. In both the global and local zero-back tasks, participants were asked to count the number of times “S” appeared on the screen (ranging between 30 and 60 times). In the two-back global and local tasks, participants were asked to count the number of times the current letter (at the appropriate level) is the same as the one presented two trials before the current trial. The range of occurrences was similar for both the zero-back and two-back tasks. At the end, participants were asked the following on a sheet of paper: (1) Please rate the cell phones shown (*X*1, *X*2, *X*3, *X*4) on a scale of 0–100 where 0 is worst and 100 is best. (2) Which is the best cell phone according to you? (3) Try to recollect as many attributes you can and state whether they were present (+) or absent (−) for each of the cell phones. Note that the participants did not know that they would be asked a question related to memory so that memory-based processes (Lassiter et al., 2009) and unconscious thought processes are not conflated.

### Results and discussion

Data from seven participants had to be rejected since they had chosen an item but rated another as the best and two participants were rejected due to very poor performance in the two-back task. In our study, we tried to keep the task difficulty and performance in the global and local tasks to be close to each other so that the differences in global and local conditions can be attributed to differences in scope of attention and not to task difficulty *per se*. In terms of performance in the global-local zero-back task, participants were accurate in both the global and local conditions with a mean error of 1.9 and 1.3% respectively. In the two-back task, the error rates in the global and local conditions were 37.18 and 38.31% respectively. We obtained a Bayes factor of 0.11 for the performance difference between global and local zero-back tasks providing strong evidence for the null (Dienes, 2008; Rouder et al., 2009). With the two-back global and local tasks, we obtain a Bayes factor of 0.57 indicating that sufficient evidence is not present to reject the null hypothesis. The results suggest that there is no difference in task difficulty between the corresponding global and local zero-back and two-bask task conditions. This suggests the task difficulty for the global and local tasks are similar for a given working memory load.

Chi-square analysis of the choices made showed that there was no significant difference in the actual choice made between the global and local conditions in both the zero-back and two-back tasks respectively. Based on the conflicting results obtained in UT studies and lack of a reliable effect even when conscious thought was compared to UT (Newell et al., 2009), it is not surprising that we did not obtain a reliable difference for the choice made when the nature of distraction task was manipulated.

**Figure 2:**
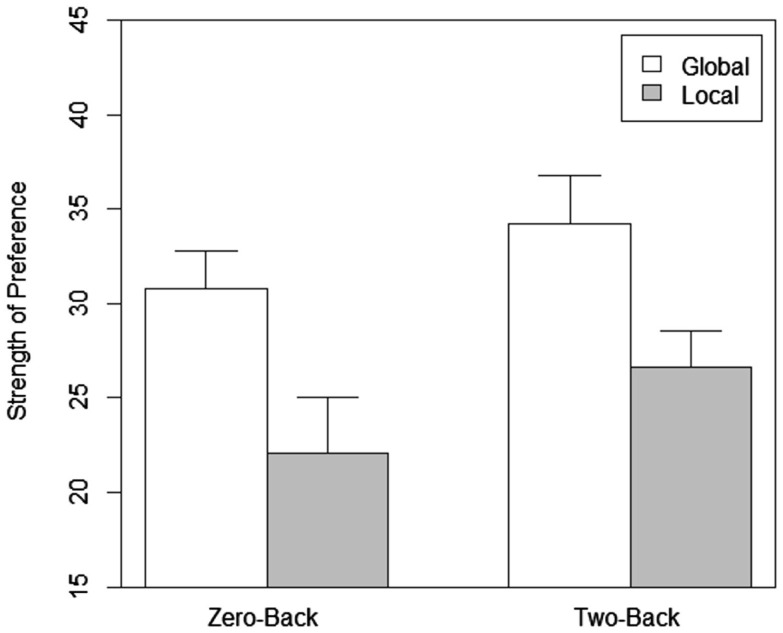
**Strength of preference as a function of scope of attention and load in Experiment 1**.

We performed a two variable between-subjects ANOVA with scope and load as the variables on strength of preference, memory for all attributes, and memory for attributes of the preferred choice. Strength of Preference was calculated by subtracting the average rating of the three least rated cell phones from the one rated highest (Dijksterhuis and van Olden, 2006; Lassiter et al., 2009). Results for preference strength and memory for both the global and local conditions in the two different load conditions are shown in Figure 2. As predicted, the results showed a significant main effect for scope, *F*(1, 76) = 11.881, *p* = 0.001 with better strength of preference for global compared to local distractor task conditions. The effect of load (*p* = 0.117) and interaction between load and scope (*p* = 0.794) was not significant. Planned comparisons showed that participants performing the global zero-back task had significantly higher strength of preference than those who performed the local zero-back task, *t*(38) = 2.7, *p* = 0.011^1^. Moving to the two-back task, the global condition once again had significantly better strength of preference, *t*(38) = 2.17, *p* = 0.036 compared to the local condition. The difference in strength of preference between the low and high load tasks for the global, *t*(38) = 0.972, *p* = 0.337 and local conditions, *t*(38) = 1.417, *p* = 0.165 was not significant.

**Figure 3:**
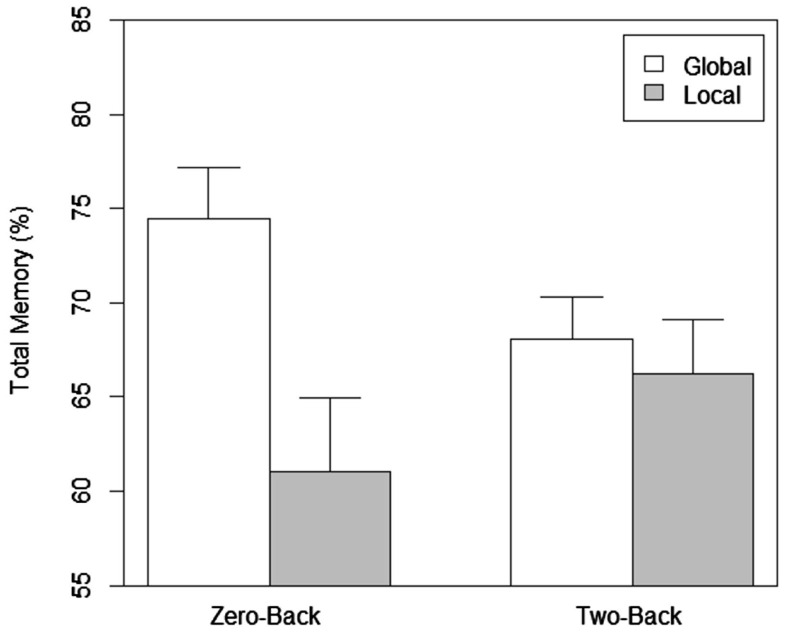
**Total memory as a function of scope of attention and load in Experiment 1**.

**Figure 4:**
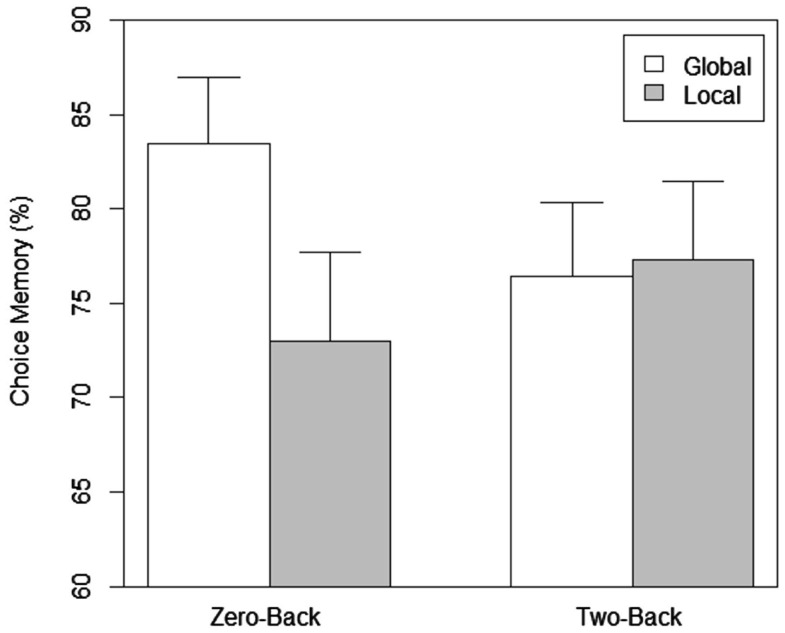
**Choice memory as a function of scope of attention and load in Experiment 1**.

In terms of total memory performance (see Figure 3) with the zero-back, the main effect of scope was significant, *F*(1, 76) = 6.474, *p* = 0.013. The effect of load was not significant (*p* = 0.834). The interaction between load and scope was close to significance, *F*(1, 76) = 3.705, *p* = 0.058. The analysis with the memory for attributes of the chosen item showed that the main effects of load (*p* = 0.734) and scope (*p* = 0.247) was not significant. The interaction between load and scope was not significant (*p* = 0.168). Planned comparisons show that the percentage of correct entries was significantly higher in the global compared to the local zero-back task, *t*(38) = 2.83, *p* = 0.007. The difference between the percentage of correct entries for the chosen cell phone (preferred choice memory) for the global and local zero-back tasks was also statistically significant, *t*(38) = 1.99, *p* = 0.05. We also compared the percentage of correct entries for all the items with that for the preferred choice between corresponding scope and load conditions. Enhanced memory performance for the chosen item (see Figure 4) was statistically significant in both the global, *t*(19) = 2.663, *p* = 0.015 and local, *t*(19) = 2.884, *p* = 0.009 zero-back task conditions. With the more difficult two-back task conditions, there was no significant difference in total and preferred choice memory performance between the global and local conditions (*p* > 0.5). The difference in total memory performance between the low and high load tasks for the global, *t*(38) = 1.82, *p* = 0.08, and local conditions, *t*(38) = 1.06, *p* = 0.296 was not significant. The difference in choice memory performance between the low and high load tasks for the global, *t*(38) = 1.205, *p* = 0.206, and local conditions, *t*(38) = 0.757, *p* = 0.454 was also not significant. However, the memory for attributes of the chosen item compared to total memory was better in the two-back global, *t*(19) = 2.203, *p* = 0.04, and local, *t*(19) = 3.502, *p* = 0.002 conditions.

The strength of preference was clearly better with global compared to the local task conditions. The effect was present under both the easy (low working memory load) and difficult (high working memory load) conditions of the distraction task and thus load itself did not have an effect on strength of preference. The effect of scope of attention on preference may be due to the link between (low-approach) positive motivational or emotional stimuli (Gable and Harman-Jones, 2010) and distributed attention (Fredrickson and Branigan, 2005; Srivastava and Srinivasan, 2010; Srinivasan and Gupta, 2011). In addition, as distributing attention or global processing is linked to an approach motivation (Förster and Higgins, 2005) and greater likings for atypical objects (Förster and Denzler, 2012a), it appears that by distributing attention, preference strength for the chosen alternatives increases as we had previously hypothesized.

One may try to argue that the difference in preference due to scope of attention might also be due to task difficulty with local processing being possibly more difficult than global processing (Navon, 1977). If task difficulty is the critical factor, then one would expect that the strength of preference for the two-back task to be much lower than the values for the zero-back conditions according to the classical understanding as more difficult tasks should cause more interference in processing. We did not find an effect for task difficulty on preference strength although the low load task was much easier than the high load task. While the difference between corresponding global and local tasks under different load conditions was not significant, the values were actually a bit higher with the more difficult two-back task conditions compared to the zero-back conditions. This needs to be contrasted with the difference between global and local conditions in which preference for the global task is more than that of the local task. The results indicate that the effect on strength of preference is not due to task difficulty *per se* but due to differences in scope of attention. The nature of the difficulty of the distractor task did not matter and if at all there is a trend, difficulty appears to increase preference strength rather than decrease it.

Unlike the effects on the strength of preference, the memory performance shows that the effect of scope of attention is probably modulated by load. Memory is better for global compared to local only when a low load easier (zero-back) distractor task is performed and enough resources are available. With the more difficult high load task, scope does not matter. A plausible explanation for better memory is that distributed attention during distraction enables selection of more items either during distraction or retrieval but only when enough cognitive resources are available. These results clearly indicate that the type or scope of attention *during* distraction significantly affects processes associated with both choice preference and memory but the influence differs based on the availability or non-availability of attentional resources (not) utilized by the distractor task.

The differences in the influence of scope in the context of different amounts of load indicate that the effects on preference and memory are driven through different mechanisms. In comparison to focused attention, the effects of distributed attention on preference and memory can be attributed either to selection of items during distraction (or UT), or to the lingering effects of attentional strategies during retrieval but enough resources are needed for the differing attentional mechanisms to influence memory of attributes. The results do clearly indicate that the preference effects are due to differences in the scope of attention between global or local processing given the fact that the more difficult two-back task did not seem to decrease preference at all (it is to be noted that the accuracy of the global and local tasks in our study are similar).

## Experiment 2

One issue that has been debated in the context of UT and decision making is whether the processes involved occur during and after the distracter task (off-line) or during the presentation of attribute information (online) before the distracter task (Lassiter et al., 2009; Waroquier et al., 2010; Strick et al., 2010). If the decision is taken during attribute presentation stage itself, then decision processes (and preference strength) could be influenced by performing the global-local task before encoding. Given that both preference and memory effects for scope of attention are present only for the zero-back task condition, we focus primarily on the low load zero-back task condition in this experiment. In addition, previous studies have shown that performing a more difficult task like the ones that require exertion of self-control results in reduction of efficiency of control processes (Baumeister et al., 2007). The memory effect due to scope of attention under low load may be more general. We hypothesized that if a global task results in larger selection of attribute information then it could enhance memory and thereby suggest that scope of attention affects encoding itself. Hence, in our second experiment a global-local task was assigned to the participants prior to giving them any information about the choice alternatives. Upon completion of the task, participants were presented with choice alternatives, asked to rate the cell phones immediately, and probed for memory of the attributes, as in Experiment 1. We used only the zero-back task since both preference and memory effects were obtained only in the low load condition in Experiment 1.

### Method

#### Participants

Forty naïve adults with normal or corrected-to-normal vision from Allahabad were selected for this study, and paid a nominal amount for participating in the experiment.

#### Stimuli and apparatus

All the stimuli and apparatus were the same as in Experiment 1.

#### Procedure

Participants were first assigned a global or a local zero-back task. They were then presented with attribute information for the four cell phones, as in Experiment 1a (see Figure 1). After information presentation, participants were asked to immediately rate all the four cell phones, choose the best cell phone (immediate mode of thought according to UTT) and finally, they were asked to recall the attribute information.

### Results and discussion

**Figure 5:**
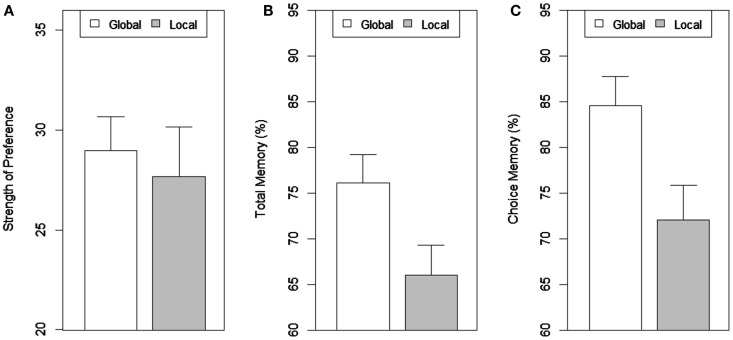
**(A)** Strength of preference, **(B)** total memory, and **(C)** choice memory as a function of scope of attention in Experiment 2.

Data from one participant was excluded from the analysis because of the presence of an outlier (>2.5 standard deviation) in the total memory measure^2^. As in Experiment 1, the performance in the global and local tasks was accurate, with non-significant error percentages of 0.76% and 1.8% respectively. The Bayes factor was 1.18 which does not provide sufficient evidence to reject the null hypothesis (no difference in performance between global and local tasks). Chi-square tests indicated no significant difference in the actual choices made in the global or local conditions. Strength of preference and memory performance for all the items as well as the preferred item in Experiment 2 is shown in Figure 5. The strength of preference was not significantly different between the global and the local task groups, *t*(37) = 0.415, *p* = 0.68^3^. However, the percentage of total correct entries recalled was significantly more for the global task group compared to the local task group, *t*(37) = 2.213, *p* = 0.033. In addition, the percentage of correct entries for the chosen item recalled accurately was also significantly more for the global compared to the local group, *t*(37) = 2.49, *p* = 0.017. The memory for attributes of the chosen item compared to all the items was better in the global condition, *t*(18) = 3.174, *p* = 0.005 but not significantly different in the local condition, *t*(19) = 1.43, *p* = 0.169.

Our results clearly demonstrate that global-local processing before encoding of attribute information about choices affects memory. These findings suggest that the type of attention primes information acquisition. Distributed attention resulted in selection of more items that resulted in better memory possibly because attended items get comparatively more access (Vogel and Machizawa, 2004; McElree, 2006). However, unlike Experiment 1, the scope of attention did not affect preference strength. This indicates that some amount of time might be needed for building preference for a particular choice specifically during distraction that could be influenced by differences in scope of attention associated with the distractor task. The results do clearly indicate that the processes underlying the effect of attention on preference and memory are possibly different. Taken together the results of Experiment 1 and 2 indicate that at least the effect on choice preference is due to the manipulation of the type of attention during distraction (UT) and hence not all decision making processes are purely online (or completed during information acquisition). It is also possible that a distraction period serves an updating role on previous choice processes.

## General Discussion

The results of both the experiments show that scope of attention affects memory and preference. However, the effects of scope of attention on these two factors differ. These results suggest that the effects of attentional scope on preference strength and memory may be mediated by different processes, and a delay or distraction is necessary in between information presentation and the choice for preference strength but not for memory. The effect of scope of attention on strength of preference was seen only in Experiment 1, where attention was manipulated during distraction. The lack of effect on preference in Experiment 2 suggests that a time interval after encoding is necessary to affect preference strengths triggered by attention strategies during distraction. Furthermore, better memory alone is not sufficient to drive preference strength: globally primed participants have better memory although strength of preference remains unaffected, in Experiment 2. The positive effect of global attention during distraction on preference strength for the chosen item may be due to the association between global processing and positive emotions (Srinivasan and Hanif, 2010; Srinivasan and Gupta, 2011) and/or an approach orientation (Förster and Higgins, 2005). Scope of attention influenced preference but task difficulty *per se* (in terms of load) did not influence preference. The fact that there is no decrease in preference (trend was in the opposite direction) as a function of task difficulty but there is a difference between global and local attention indicates that the effect on preference is likely due to the motivational or emotional differences associated with differences in scope of attention (Förster and Higgins, 2005; Srinivasan and Hanif, 2010; Srinivasan and Gupta, 2011).

Earlier studies on memory have linked larger scope of attention to larger working memory capacity and better cognitive processing (Cowan, 2005; Cowan et al., 2006) that presumably enable selection of a larger number of items and better processing of selected items (Vogel and Machizawa, 2004). We show that the scope of attention can be manipulated using a perceptual task that can consequently alter memory performance for the attributes of given choices when sufficient resources are available. In addition, the amount of resources utilized by the distractor task as a function of task difficulty is important for the effect of attentional scope on memory with global attention enhancing memory only under low load conditions. As evident from Experiment 2, when the scope of attention is induced by an easier global-local task, the effects persist for at least a few minutes.

Our findings have significant implications on UTT. The results of both our experiments suggest that studies on *directing attention away* from the decision making task are far from complete. Scope of attention *during* distraction affects both preference formation and memory, but preference irrespective of task difficulty and memory when the distractor task is an easy task. Previous studies have paid little attention to the kind of processing actually performed during UT, assuming it simply to be a way of directing attention elsewhere. However, the results of our studies clearly show that the kind of processing performed during distraction affects the nature of processes involved in our judgments on affective quality and memory. We agree with Simonson (2005) that the manipulations spearheaded by Dijksterhuis and colleagues can shed light in various real life scenarios like consumer decisions. However, we feel that there is an urgent need to investigate the processes that occur during UT (perhaps as a function of the distractor task itself) rather than argue over whether unconscious or conscious thought is better.

Attention is not unitary and cannot be simply treated as a dichotomous variable that may or may not be present in thought processes. Manipulation of the scope of attention by global and local processing can produce different effects on the decision about an unrelated item. While attentional mechanisms might differ between conscious and unconscious thought, the definition of unconscious thought as “thought without attention or with attention directed elsewhere” as proposed by Dijksterhuis and Nordgren (2006), is probably *incomplete:* UT itself is affected by consciously controlled attentional strategies. The concept of “UT” as thought without attention ignores the fact that attentional processes themselves can differ.

Our results suggest that while scope of attention manipulated either before encoding or during distraction improves memory (but only in the context of the low load task), broad scope of attention increases preference only during distraction. According to UTT, explicit memory of attributes and the choice strength need not be positively correlated as the “unconscious” should lead to automatic weighting. However, we found that following a period of distraction in the global task condition, participants had greater preference strength for the choice. This observation raises the question on whether distraction is essentially “*distracted conscious thought*” rather than “*unconscious thought*.” To our knowledge, there is no evidence yet that distracting attention via an unrelated task as in typical UT studies promotes “unconscious” processing (also see Aczel et al., 2011). The operationalization of unconscious thought slips from using the resource view of attention to comment on consciousness, and UT theorists have conflated both attention and consciousness in the process (Srinivasan and Mukherjee, 2010). We know that attention and consciousness are dissociable as distinct processes (Lamme, 2003; Srinivasan, 2008; van Boxtel et al., 2010), and therefore the individual roles of each in decision making requires more critical evaluation.

Instead of arguing whether unconscious or conscious thought results in better memory consolidation (Dijksterhuis et al., 2006; Shanks, 2006), we show the necessity of comparing different types of pre- and post-information acquisition periods on memory for the attributes. Both the global and local task can be conceived as periods of “thinking away from the problem”: the memory effects produced vary with the types of distractions or UT tasks. Although we do not argue that decisions are taken online or off-line, our results show that the affective quality of judgments is definitely affected by both distraction/UT and processes during information presentation. These findings suggest complementary roles for both online and off-line mechanisms.

Further studies about the nature of processing before and after information-encoding would go a long way in fully understanding the way we make complex decisions. For example, it is important to consider the mechanisms involved in preference strength generation and examine the mediating roles of explicit and implicit memory on preference constructions. We also need to understand the temporal dynamics of both choice preference stabilizations and the effects of attentional scope on memory retrieval. Moreover, as pointed out earlier, it is possible that memory and preference can be mediated and facilitated by different cognitive effects. We agree with Nordgen et al. (2011), who suggested that complex decisions are a product of interrelated mechanisms including possible integration of conscious and unconscious modes. We believe that processes differing in attentional mechanisms, memory and heuristics are based on an underlying continuum in decision making.

It is important to recognize that scope of attention is a fundamental factor that can influence various types of daily decision making. To our knowledge, this is one of the first studies to show that global versus local perception can affect consumer decision related processes, especially preference strength in consumer judgments. However, our study is only suggestive of the whole range of questions concerning the role of attention in decision making. Thus far, the role of attentional scope is relatively obscure in theoretical treatments of judgment and decision making. Narrow versus wide scope as measured by global versus local perceptual tasks can affect both “regulatory focus” or the way we approach a problem (Förster and Higgins, 2005) and the later judgments we form (Liberman and Förster, 2009; Förster and Denzler, 2012a). Scope of attention might also interact with the amount of attention during a choice problem. Further studies investigating scope versus amount of attention could help us understand newer intricacies of processing different modes of thought that, in turn, will shed more light on the role played by attention in decision making. More importantly, decision making also needs to be viewed in the light of attentional scope or strategies instead of only treating attention as a resource that aids selection of information.

## Conflict of Interest Statement

The authors declare that the research was conducted in the absence of any commercial or financial relationships that could be construed as a potential conflict of interest.

## Footnotes

^1^We also performed Bayesian analysis (Rouder et al., 2009) and computed the Unit Bayes factor (BF) for the three dependent measures (with *r* = 1) comparing the differences between global and local processing conditions under low load and high load conditions. All the BF values reported in the paper are evidence for the alternative with respect to the null hypothesis. High BF values (much greater than 1) indicate evidence for the alternative hypothesis and low BF values (much less than 1) indicate evidence for the null hypothesis. The BF for the strength of preference difference between global and local processing conditions was 6.62 providing strong support for the alternative hypothesis, that strength of preference is different in the global compared to the local conditions. The BF for the difference in strength of preference between global and local tasks with the two-back task was 2.37. In terms of total memory performance with the zero-back, the BF for the difference between the global compared to the local task was 9.09. The BF for the difference between preferred choice memory for the global and local zero-back tasks was 1.733. With the more difficult two-back task conditions, the BF for the difference in total and preferred choice memory performance between the global and local conditions was 0.339 and 0.305 respectively.

^2^The outlier participant had values of 31.25% for attributes of all items (total memory) and 50% for memory of attributes for the chosen item. Statistical analysis performed by retaining the outlier makes the effect of scope of attention on total memory non-significant, *t*(38) = 1.576, *p* = 0.12 with the trend remaining the same (global = 73.8%, local = 66%). The effect of scope of attention on memory for attributes of the chosen item remains significant, *t*(38) = 2.08, *p* = 0.04.

^3^The BF for the strength of preference between the global and the local task groups was 0.330 indicating strong evidence for the null, that is there is no difference in strength of preference between the global and local conditions. The BF for the percentage of total correct entries in the global task group compared to the local task group was 2.565. The BF for the preferred choice memory in the global task compared to the local task condition was 4.316 indicating a strong evidence that memory is better in the global compared to the local group.
